# Low Plasma Levels of Irisin Predict Acutely Decompensated Heart Failure in Type 2 Diabetes Mellitus Patients with Chronic Heart Failure

**DOI:** 10.3390/jcdd10040136

**Published:** 2023-03-23

**Authors:** Alexander A. Berezin, Anica Babic Obradovic, Ivan M. Fushtey, Tetiana A Berezina, Michael Lichtenauer, Alexander E Berezin

**Affiliations:** 1 Department of Internal Medicine, Zaporozhye Medical Academy of Postgraduate Education, 69096 Zaporozhye, Ukraine; 2 Medical Practice for General Medicine, 22111 Hamburg, Germany; 3 Vita Center, Department of Internal Medicine and Nephrology, 69000 Zaporozhye, Ukraine; 4 Division of Cardiology, Department of Internal Medicine II, Paracelsus Medical University, 5020 Salzburg, Austria; 5 Department of Internal Medicine, Zaporozhye State Medical University, 69035 Zaporozhye, Ukraine

**Keywords:** acutely decompensated heart failure, chronic heart failure, type 2 diabetes mellitus, irisin, natriuretic peptides, cardiac remodeling

## Abstract

The aim of this study was to determine the discriminative value of irisin for acutely decompensated heart failure (ADHF) in type 2 diabetes mellitus (T2DM) patients with chronic HF. We included 480 T2DM patients with any phenotype of HF and followed them for 52 weeks. Hemodynamic performances and the serum levels of biomarkers were detected at the study entry. The primary clinical end-point was ADHF that led to urgent hospitalization. We found that the serum levels of N-terminal natriuretic pro-peptide (NT-proBNP) were higher (1719 [980–2457] pmol/mL vs. 1057 [570–2607] pmol/mL, respectively) and the levels of irisin were lower (4.96 [3.14–6.85] ng/mL vs. 7.95 [5.73–9.16] ng/mL) in ADHF patients than in those without ADHF. The ROC curve analysis showed that the estimated cut-off point for serum irisin levels (ADHF versus non-ADHF) was 7.85 ng/mL (area under curve [AUC] = 0.869 (95% CI = 0.800–0.937), sensitivity = 82.7%, specificity = 73.5%; *p* = 0.0001). The multivariate logistic regression yielded that the serum levels of irisin < 7.85 ng/mL (OR = 1.20; *p* = 0.001) and NT-proBNP > 1215 pmol/mL (OR = 1.18; *p* = 0.001) retained the predictors for ADHF. Kaplan–Meier plots showed a significant difference of clinical end-point accumulations in patients with HF depending on irisin levels (<7.85 ng/mL versus ≥7.85 ng/mL). In conclusion, we established that decreased levels of irisin were associated with ADHF presentation in chronic HF patients with T2DM independently from NT-proBNP.

## 1. Introduction

Acutely decompensated heart failure (ADHF) is defined as rapidly progressive pre-existing cardiomyopathy often due to the dysregulation of neuro-humoral adaptive mechanisms, which act to maintain hemodynamic and perfusion of target organs despite worsening cardiac function [1]. The patients with ADHF demonstrate a high variability of signs and symptoms of congestion and fluid retention which, in the majority of cases, lead to urgent hospital admission [2,3]. ADHF continues to be associated with unacceptably increasing in-hospital mortality rates (7.5%) and one-year mortality rates (20.1–23.3%) [4]. Although a short-term prognosis of ADHF remains to be poor, hemodynamically stable patients after ADHF continue to be at higher risk of unfavorable post-discharge clinical outcomes [5,6]. Indeed, 30-day HF readmission rates were 4.8–5.4%, one-year HF readmission average rates were from 23.6% to 26.2%, and the 60-day rate of readmission or cardiovascular (CV) death is between 31% and 50% [4,5,6,7].

Distinct scenarios of the natural course of ADHF relate to clinical heterogeneity among patients admitted to hospitals, cardiac dysfunction etiology, precipitating factors contributing to heart failure (HF) decompensation, and the current implementation of guideline-based medical therapy [8,9,10]. Euro Heart Failure Survey II revealed that 40% of ADHF patients had type 2 diabetes mellitus (T2DM) and that almost half of individuals exerted multiple co-morbidities including atrial fibrillation (AF), chronic kidney disease (CKD), hypertension, and coronary artery disease [11]. Moreover, the majority of ADHF patients had polypharmacy and variable side effects of medications [12].

Despite several factors, such as clinical phenotypes of patients with known HF, phenotypes of HF, comorbidity profile, natriuretic peptides (NPs), and a personally adjusted care program for HF, short- and long-term clinical outcomes do not seem to be concisely predicted [13,14]. Yet, a lung ultrasound and echocardiography with measurements of cardiac features including left ventricular ejection fraction (LVEF) and diastolic dysfunction parameters were the most useful tools for affirming the presence of ADHF, but not for predicting the condition [15]. In addition, NPs were more valuable in excluding ADHF than in the prediction of the occurrence of the disease in patients with any phenotype of HF [16]. Indeed, despite the utilization of NPs as routine, costly, affordable, easy-to-use tests for an HF diagnosis in emergency departments, their predictive potency for ADHF appears to be sufficiently variable and dependent on the HF phenotype and T2DM presence [17]. In this context, there is a need to discover new approaches to identify chronic HF patients with concomitant T2DM at a higher risk of ADHF depending on their comorbidity status [18].

Irisin is a multifunctional peptide, which is proteolytically cleaved from its precursor fibronectin type III domain-containing protein 5 which is mainly secreted by skeletal muscles and cardiac myocytes [19,20]. Irisin plays a crucial role in energy homeostasis and regulates glucose metabolism, insulin sensitivity, and mitochondrial oxidation of free fatty acids [21]. Therefore, irisin maintains cardiac function and prevents cardiac injury, cardiac myocyte necrosis and apoptosis, extracellular matrix remodeling, and inflammatory reaction [22]. Yet, irisin seems to show a cardiac protective effect in T2DM patients with chronic HF treated with SGLT2 inhibitors [23]. Previously, it has been reported that low levels of irisin predicted mortality risk in acute HF patients [24] and chronic HFrEF/HFpEF [25,26]. However, the role of irisin in predicting ADHF in T2DM patients with chronic HF remains as not fully understood. The aim of the study is to determine the discriminative value of irisin for ADHF in T2DM patients with chronic HF.

## 2. Materials and Methods

### 2.1. Study Design and Cohorts of Participants

**Figure 1:**
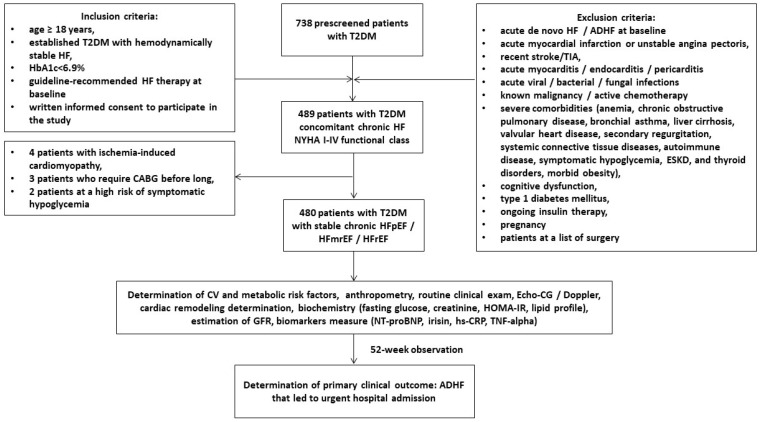
Flow chart of the study design. Abbreviations: ADHF, acutely decompensated heart failure; CV, cardiovascular; Echo-CG, echocardiography; GFR, glomerular filtration rate; HF, heart failure; HFpEF, heart failure with preserved ejection fraction; HFmrEF, heart failure with mildly reduced ejection fraction; HFrEF, heart failure with reduced ejection fraction; hs-CRP, high sensitivity C-reactive protein; HOMA-IR, Homeostatic Assessment Model of Insulin Resistance; HbA1c, glycosylated hemoglobin; NT-proBNP, N-terminal brain natriuretic pro-peptide; T2DM, type 2 diabetes mellitus; TIA, transient ischemic attack; and TNF-alpha, tumor necrosis factor-alpha.

A total of 738 patients with T2DM were prescreened using the local database of “Vita Center” (Zaporozhye, Ukraine). Using criteria of inclusion (male/female with age of ≥18 years, established T2DM with hemodynamically stable chronic HF, glycosylated hemoglobin < 6.9%, informed consent to participate in the study), we enrolled 489 patients with T2DM with concomitant chronic HF I-IV New York Heart Association (NYHA) functional classes (Figure 1). The exclusion criteria were acute de novo HF, acute coronary syndrome/myocardial infarction or unstable angina pectoris, recent stroke/transient ischemic attack, acute myocarditis/endocarditis/pericarditis, known malignancy and/or chemotherapy, acute viral/bacterial/fungal infections, severe co-morbidities (anemia, chronic lung and liver diseases, known inherited and acquired heart defect, symptomatic severe hypoglycemia, morbid obesity, systemic connective tissue diseases, autoimmune disease, cognitive dysfunction, and thyroid disorders), pregnancy, type 1 diabetes mellitus, or current therapy with insulin. Then, we excluded nine patients who were not able to be under continuous monitoring for 52 weeks. Finally, we selected in the study 480 patients with T2DM with any phenotype of chronic HF who were followed from June 2021 to August 2022. During the 52-week observation period, we pooled patient data from different sources including physician records, databases, discharge reports, autopsy reports, and direct calls to patients and/or their relatives.

### 2.2. Determination of Study End-Points

The primary clinical end-point was ADHF that led to urgent hospitalization. ADHF was defined as the clinical presentation of signs and symptoms of congestion (elevated jugular venous pressure, orthopnea, bilateral leg edema, pulmonary rales, third heart sound, pulmonary edema on chest X-ray, nocturnal cough, dyspnea with exertion, recent diuresis, and onset of hepatomegaly and/or pleural effusion) [1].

### 2.3. Concomitant Medical Information Collection

Basic clinical data, including age, gender, height, weight, waist circumference, hip-to-waist ratio (WHR), body mass index (BMI), and body surface area (BSA) comorbidities (hypertension, T2DM history, and dyslipidemia), and smoking were collected. T2DM was established according to the conventional criteria provided by the American Diabetes Association [27]. The European Society of Cardiology (ESC) clinical guidelines were used to detect HF [1], hypertension [28], dyslipidemia [29], and stable coronary artery disease [30]. Chronic kidney disease in T2DM patients was established in accordance with the Kidney Disease Improving Global Outcomes (KDIGO) Consensus Report [31].

### 2.4. Echocardiography Examination

Enrolled patients underwent transthoracic B-mode echocardiography and Doppler examination, which was performed by a blinded high qualified ultra-sonographer using the diagnostic system Vivid T8 (“General Electric Medical Systems”, Freiburg, Germany) Cardiac volumes including left ventricular end-diastolic (LVEDV) and end-systolic (LVESV) volumes and left atrial volume (LAV) were measured in the standard apical 4-chamber view in compliance with current guidelines [32,33]. The LAV index (LAVI) was calculated as a ratio of LAV to BSA. Left ventricular (LV) ejection fraction (LVEF) was estimated by the Simpson method. Diastolic parameters included early diastolic blood filling (E), longitudinal strain ratio (e`), and their ratio (E/e`) at baseline and at the 52nd week of the follow-up. Estimated E/e` ratio was expressed as the ratio equation of E wave velocity to averaged medial and lateral e’ velocity [33]. Left ventricular hypertrophy (LVH) was detected according to conventional recommendations [33], which used LV myocardial mass index (LVMMI) ≥ 125 g/m^2^ or ≥110 g/m^2^ in males and females, respectively, as a marker of LVH.

### 2.5. Blood Sampling and Biomarker Measurements

Fasting blood samples from patients were collected from an antecubital vein (3–5 mL) and maintained at 4 °C at baseline and in the 52-week interval of the follow-up. After centrifugation (3000 r/min, 30 min), polled serum aliquots were immediately stored at ≤−70 °C until analysis. Serum concentrations of NT-proBNP, irisin, tumor necrosis factor-alpha (TNF-alpha), and high-sensitivity C-reactive protein (hs-CRP) were determined using commercially available enzyme-linked immunosorbent assay (ELISA) kits (Elabscience, Houston, TX, USA) according to the manufacturer’s instructions. Both the intra- and inter-assay coefficient of variability for each biomarker were <10%. Conventional biochemistry parameters were routinely measured at the local biochemical laboratory of Vita Center (Zaporozhye, Ukraine) using a Roche P800 analyzer (Basel, Switzerland).

### 2.6. Estimation of Glomerular Filtration Rate

We used CKD-EPI formula to estimate the glomerular filtration rate (GFR) [34].

### 2.7. Determination of Insulin Resistance

Insulin resistance was evaluated using the Homeostatic Assessment Model of Insulin Resistance (HOMA-IR) [35].

### 2.8. Statistics

V. 23 Statistical Packages for Social Sciences (SPSS; IBM, Armonk, New York, NY, USA) software and v. 9 GraphPad Prism (GraphPad Software, San Diego, CA, USA) software for statistical analysis were used. Normal distribution of variables was evaluated with the Kolmogorov–Smirnov test. Mean (M) ± standard deviation (SD) and median (I) and 25–75% interquartile range (IQR), respectively, characterized continuous normally and non-normally distributed variables. The difference between categorical values was assessed with the chi-square test. Student’s *t*-tests or one-way analyses of variance (ANOVA) with the Mann–Whitney U test were used for the comparison between groups depending on variable distribution. Spearman’s correlation coefficient was calculated to ascertain the relationship between variables. Receive Operation Curve (ROC) curves with a separate analysis of the Youden Index were performed to assess the reliability of predictive models. Predictors for ADHF were determined by a univariate logistic regression. All variables with *p* < 0.1 were entered in a backward stepwise multivariate logistic regression analysis and then variables with the highest *p* value were eliminated from the whole model. The selection was stopped when the *p* value was smaller than the pre-specified threshold determined by Bayesian information criterion. An odds ratio (OR) and 95% confidence interval (CI) were reported for each predictor. Predictors of ADHF were confirmed using integrated discrimination indices (IDI) and net reclassification improvement (NRI). Kaplan–Meyer curve analysis was used with the aim of elucidating plausible benefit in clinical outcome occurrence depending of irisin cutoff levels (≥7.85 ng/mL versus <7.85 ng/mL). The intra-class correlation coefficient was used to determine both inter- and intra-observer reproducibility for irisin levels and echocardiographic parameters from 50 randomly selected HF patients using an identical cine-loop for each view. Differences were considered significant at the level of statistical significance *p* < 0.05.

## 3. Results

### 3.1. Basis Characteristic of the Patients Enrolled in the Study

The entire patient population includes male and female (56.7% and 43.3%, respectively) with a mean age of 53 years (Table 1). The average BMI was 25.6 ± 2.78 kg/m^2^, waist circumference was 96.7 ± 3.90 cm, and WHR was 0.88 ± 0.07 units. The patients had several cardiovascular and metabolic risk factors and diseases, such as dyslipidemia (80.2%), left ventricular hypertrophy (79.5), hypertension (64.0%), obesity (44.8%), stable coronary artery disease (34.6%), chronic kidney disease 1–3 grades (24.6%), atrial fibrillation (21.9%), smoking (40.8%) and dilated cardiomyopathy (4.0%). HF phenotypes were qualified as the following: HFpEF (44.2%), HfmrEF (32.5%), and HFrEF (23.3). All patients were hemodynamically stable and 58.6% of them had I/II HF NYHA class, 30.0% of patients exhibited III HF NYHA class, and 11.4% of individuals had IV HF NYHA class. The average LVEF was 45% (34–57%) and mean value of LAVI was 41 mL/m^2^ (33–52 mL/m^2^). Fasting glucose, creatinine, and HbA1c were 6.20 ± 1.2 mmol/L, 98.7 ± 9.8 µmol/L and 6.40 ± 0.14%, respectively. The serum levels of hs-CRP and TNF-alpha were 4.20 mg/L (2.51–7.10 mg/L) and 2.95 pg/mL (1.66–3.82 pg/mL), respectively. The average NT-proBNP and irisin in serum were 1215 pmol/mL (562–2155 pmol/mL) and 5.64 ng/mL (3.80–7.53 ng/mL), respectively. All patients received guideline-recommended therapy of HF and antidiabetic agents.

There were no significant differences between the groups in the demographic and anthropomorphic parameters, presentation of dyslipidemia, hypertension, smoking, abdominal obesity, left ventricular hypertrophy, HFpEF and HFmrEF, III HF NYHA class, as well as systolic and diastolic blood pressure, LVEDV, LVEF, or E/e`. The patients from the group with ADHF when compared with non-ADHF had frequently stable coronary artery disease (*p* = 0.024), AF (*p* = 0.001) regardless of its form, CKD 1–3 grades (*p* = 0.012), and IV HF NYHA class (*p* = 0.001). Yet, ADHF patients demonstrated higher LVESV (*p* = 0.012), LVMMI (*p* = 0.014), LAVI (*p* = 0.010), and lower eGFR (*p* = 0.026) than those without ADHF. Circulating levels of creatinine (*p* = 0.042), triglycerides (*p* = 0.044), NT-proBNP (*p* = 0.044), and TNF-alpha (*p* = 0.042) were higher in ADHF patients than in those without ADHF. Serum levels of irisin, on the contrary, were lower in ADHF than in non-ADHF patients (*p* = 0.001). Patients from both groups did not distinguish each other in concomitant medications apart from ivabradine, anticoagulants, and statins. The patients from the ADHF group had a lower frequency of ivabradine (*p* = 0.001) and statin (*p* = 0.048) prescriptions and higher anticoagulants (*p* = 0.001) use than non-ADHF individuals.

### 3.2. Determination of Primary Causes for ADHF

ADHF was associated with acute myocardial infarction in 14 patients (13.2%), a loss of control for ventricular heart rate in AF 10 patients (9.4%), malignant arrhythmia in 7 patients (6.6%), loop diuretic intolerance in 11 patients (10.3%), and a progression of chronic kidney disease in 9 individuals (8.5%). Other causes were uncontrolled hypertension (13 patients, 12.3%), T2DM (23 patients, 21.7%), transient ischemia attack/stroke (3 patients, 2.8%), pneumonia (4 patients, 3.7%), pulmonary thromboembolism (6 patients, 5.6%), and dilation cardiomyopathy (6 patients, 5.6%).

### 3.3. Clinical Features, Echocrdiographic Parameters, and Biomarkers’ Levels during the Follow-Up

A dynamic of several characteristics of the entire patient population is reported in Table 2.

In fact, in the entire group there were no significant changes in BMI, systolic and diastolic BP, cardiohemodynamic performances apart from LVESV (Δ% = −6.9%, *p* = 0.04), eGFR, fasting glucose, HbAc1, creatinine, NT-proBNP, irisin, and TNF-alpha. Along with it, hs-CRP levels were found to be increased up to 13.1% (*p* = 0.026). In the ADHF group, an elevation of creatinine, hs-CRP, and NT-proBNP were detected, whereas the levels of irisin remained lower. On the contrary, in the non-ADHF group a significant decrease in creatinine, hs-CRP, NT-proBNP, TNF-alpha, and irisin were noticed. These changes in circulating biomarkers corresponded to favorable dynamics of LVESV.

### 3.4. Spearman’s Correlation between Circulating Levels of Irisin and Other Parameters

In the entire group of patients, we found positive correlations between the levels of serum irisin and LVEF (r = 0.33; *p* = 0.001), BMI (r = 0.26; *p* = 0.012), and WHR (r = 0.24; *p* = 0.024), and an inverse correlation with NT-proBNP (r = −0.35; *p* = 0.001), NYHA class (r = 0.34, *p* = 0.001), TNF-alpha (r = −0.33; *p* = 0.001), LAVI (r = −0.32; *p* = 0.001), E/e’ (r = −0.30; *p* = 0.02), triglucerides (r = −0.24, *p* = 0.04), the HOMA index (r = −0.23, *p* = 0.01), hs-CRP (r = −0.21; *p* = 0.001), and LDL-C (r = −0.21; *p* = 0.04). There were no associations between the levels of isirin with fasting glucose, HbA1c, or HDL-C. However, there were no significant correlations between the levels of irisin with concomitant medications.

### 3.5. Discriminative Value of Irisin for ADHF

**Figure 2:**
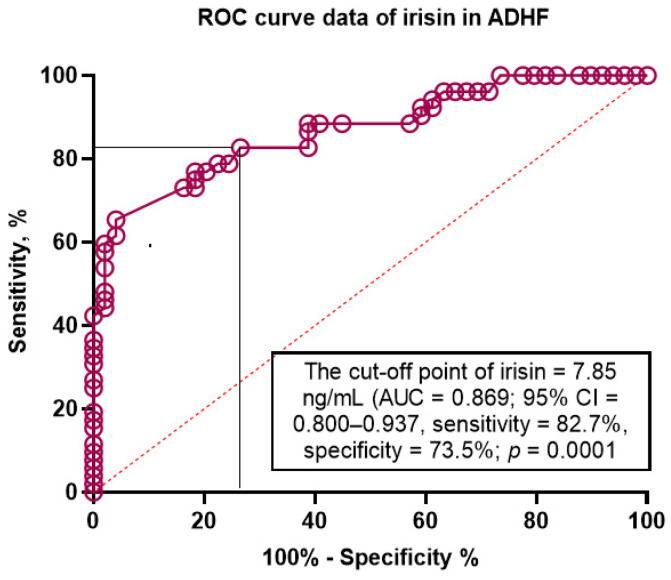
The predictive model for ADHF based on the serum levels of irisin. The results of the ROC curve analysis. Abbreviations: AUC, area under curve; CI, confidence interval.

The ROC curve analysis (Figure 2) showed that the estimated cut-off point for serum irisin levels (ADHF versus non-ADHF) was 7.85 ng/mL (area under curve [AUC] = 0.869 (95% CI = 0.800–0.937), sensitivity = 82.7%, specificity = 73.5%; likelihood ratio = 3.117; *p* = 0.0001).

### 3.6. The Predictors of HF in T2DM Patients: The Univariate and Multivariate Logistic Regression

To evaluate the predictive values of hemodynamic parameters and biomarkers, we used the cutoff point for irisin (7.85 ng/mL), as well as the median for LVEF (45%), LAVI (41 mL/m^2^), NT-proBNP (1215 pmol/mL), hs-CRP (4.2 mg/L), TNF-alpha (2.95 pg/mL), and eGFR (74 mL/min/1.73 m^2^). A univariate logistic regression showed that irisin < 7.85 ng/mL (OR = 1.24; *p* = 0.001), NT-proBNP > 1215 pmol/mL (OR = 1.16; *p* = 0.001), TNF-alpha > 2.95 pg/mL (OR = 1.06; *p* = 0.012), LV hypertrophy (OR = 1.10; *p* = 0.044), LVEF < 45% (OR = 1.09; *p* = 0.042), and LAVI > 41 mL/m^2^ (OR = 1.12; *p* = 0.026) were independent predictors for ADHF (Table 3).

The multivariate logistic regression yielded that the serum levels of irisin < 7.85 ng/mL (OR = 1.20; *p* = 0.001) and NT-proBNP > 1215 pmol/mL (OR = 1.18; *p* = 0.001) were retained as predictors for ADHF.

### 3.7. Comparison of the Predictive Models

We compared predictive models for ADHF and found that the discriminative value of irisin < 7.85 ng/mL was superior to that NT-proBNP > 1215 pg/mL, whereas there was no significant difference between Model 2 and Model 3 for ADHF (Table 4). Thus, decreased levels of irisin were associated with ADHF independently from NT-proBNP.

### 3.8. Kaplan–Meier Curve Analysis

**Figure 3:**
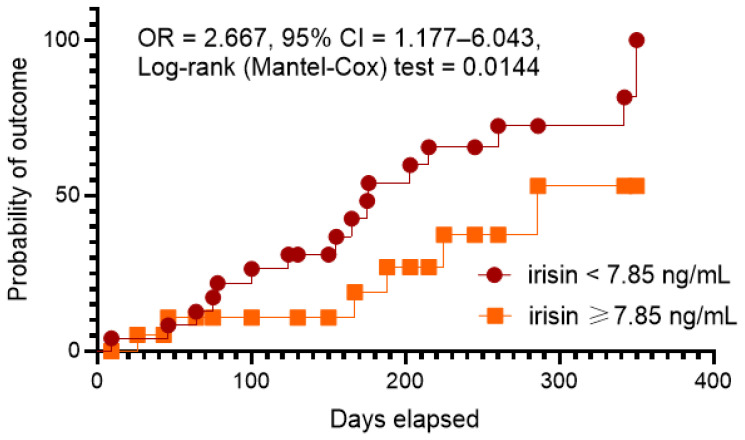
The Kaplan–Meier analysis of ADHF. Abbreviations: AUC, area under curve; CI, confidence interval; OR, odds ratio.

To confirm our hypothesis of predictive ability of low levels of irisin for ADNF, we performed the Kaplan–Meier analysis of clinical outcome. Kaplan–Meier plots showed a significant difference of clinical end-point accumulations in patients with HF depending on irisin levels (<7.85 ng/mL versus ≥7.85 ng/mL) (Figure 3). We found that patients with irisin levels ≥ 7.85 ng/mL had a benefit in ADHF occurrence when compared with those who had irisin levels < 7.85 ng/mL (OR = 2.667; 95% CI = 1.177–6.043; log rank test = 0.0144).

### 3.9. Reproducibility of Biomarkers

The evaluation of the reproducibility of irisin was performed in comparison with NT-proBNP. The intra-class correlation coefficient for the inter-observer reproducibility of NT-proBNP was 0.92 (95% CI = 0.86–0.97), whereas the intra-class correlation coefficient for intra-observer reproducibility of irisin was 0.91 (95% CI = 0.88–0.95).

### 3.10. Reproducibility of Echocardiographic Parameters

The intra-class correlation coefficient for inter-observer reproducibility of LV dimensions was 0.88 (95% CI = 0.83–0.92), of LVEF was 0.93 (95% CI = 0.90–0.97), of LAVI was 0.92 (95% CI = 0.89–0.94), and of E/e’ was 0.90 (95% CI = 0.87–0.94). Along with it, the intra-class correlation coefficient for the intra-observer reproducibility of LV dimensions was 0.91 (95% CI = 0.88–0.95), of LVEF was 0.94 (95% CI = 0.90–0.98), of LAVI was 0.95 (95% CI = 0.93–0.97), and of E/e’ was 0.92 (95% CI = 0.90–0.95).

## 4. Discussion

The results of the study revealed that the levels of irisin < 7.85 ng/mL in chronic hemodynamically stable HF patients with T2DM seem to show discriminative values for ADHF. Along with it, irisin exhibited much better predictive potency than NT-proBNP, whereas a combination of both biomarkers did not add any prognostic information for ADHF to irisin. The Kaplan–Meier curve analysis yielded sufficient benefits in clinical outcome occurrence in patients with irisin levels ≥ 7.85 ng/mL than those with <7.85 ng/mL. The majority of previous studies have reported that patients with T2DM had low levels of irisin and that irisin might be a biomarker with plausible predictive values for clinical outcomes, although there are controversial issues [17,25,26]. Indeed, Shen S. et al. (2017) [17] found that increased levels of irisin predicted mortality risk in patients with acute HF, while the authors did not especially evaluate a role of T2DM as a cofactor in the discriminative potency of the biomarker. A meta-analysis of 26 studies (number of participants = 3667) of Song R, et al. (2021) [36] showed that patients with T2DM had lower levels of irisin than healthy volunteers. In another meta-analysis of 23 studies involving 1745 diabetic patients (T2DM, T2DM, and gestational diabetes mellitus [GDM]) and 1337 non-diabetic controls, circulating irisin levels were decreased in patients with T2DM and GDM, but not in patients with T1DM [37]. Yet, an effective cardiac rehabilitation program was associated with an increase in irisin levels in peripheral blood in patients with T2DM [38]. There is strong evidence of the fact that low levels of irisin are related to reduced eGFR in T2DM patients and predicted T2DM-induced nephropathy [39]. Recently, we reported that low levels of irisin being associated with any phenotypes of chronic HF predicted poor clinical outcomes among HF patients with concomitant T2DM [25,26]. However, a link between low concentrations of irisin and a risk of ADHF in chronic HF population patients with T2DM has been detected here first.

Despite conventional clinical protocols for HF diagnosis and therapy, including a limiting number of biomarkers having validated predictive values for mainly NPs, a discovery of new biomarkers is in a loop of investigations because several selective populations of HF patients such as diabetics cannot be thoroughly stratified as at risk of HF-related complications by NPs [1,40]. Yet, there is limiting evidence for low levels of NPs in the prediction of clinical outcomes among HF patients treated with the four-pillar guideline-recommended combination, which includes the renin–angiotensin–aldosterone system mainly ARNI and MCA, beta-blocker, and SGLT2 inhibitor [41]. Irisin seems to show high reliability of its discriminative value beyond NT-proBNP for HF-related events, adverse cardiac remodeling, and mortality [42,43,44,45]. Therefore, low concentrations of irisin are found in T2DM and predict a high risk of cardiovascular complications in this population [46,47].

In our study, the main causes which contribute to the decompensation of chronic HF and led to ADHF with subsequent hospitalizations were progression of T2DM and chronic kidney disease, uncontrolled hypertension, and acute myocardial infarction. Other causes included malignant arrhythmia, loop diuretic intolerance, transient ischemia attack/stroke, pneumonia, pulmonary thromboembolism, and dilation cardiomyopathy. In fact, the majority of ADHF patients (59 individuals, 55.6%) had direct cardiovascular reasons for cardiac decompensations, whereas other patients might exhibit clinical signs and symptoms of ADHF due to numerous factors indirectly affecting cardiac injury. Therefore, uncontrolled hypertension and chronic kidney disease may be a result of an escape of glycaemia control in T2DM patients, as well as that acute myocardial infarction is a frequent complication of accelerating atherosclerosis in T2DM [48]. Thus, irisin as a multifunctional regulator of energetic homeostasis, inflammation, tissue reparation, and endothelial function is able to participate in the pathogenesis of these complications and to link cardiac remodeling in T2DM patients with a risk of ADHF [49,50,51]. Indeed, high glycemic variability, poor glycemic control, acute coronary syndromes, declining diuretic response, and uncontrolled hypertension were found to the most important factors leading to ADHF in T2DM patients with HF [52,53,54,55]. In this connection, low levels of circulating irisin, which were found in patients with T2DM, cardiovascular diseases, and chronic kidney disease in numerous previous studies [56,57,58], appear to be a promising indicator of a higher risk of ADHF regardless of the etiology of the condition.

There are numerous underlying molecular mechanisms which explain the involvement of irisin in the regulation of inflammatory response and tissue reparation with a subsequent impact on adverse cardiac remodeling, microvascular inflammation, endothelial function, kidney parenchyma survival, and browning adipose tissue [59]. Irisin acts through up-regulating the Expression of Uncoupling Protein 2 and macrophage-stimulating 1/c-Jun N-terminal kinase pathway and thereby prevents ischemia/reperfusion, suppresses inflammation, oxidative stress, and apoptosis, promotes cardiomyocyte survival, and mitochondrial homeostasis [60,61]. Yet, irisin markedly decreased the activation of the mitogen-activated protein kinase (MAPK) signaling pathway and suppressed pro-informatory cytokine expression, cellular senescence in TNF-α-stimulated cardiomyocytes, and NLRP3 inflammasome [62,63].

In our study, we did not find a significant difference between both groups of patients in the levels of hs-CRP, whereas concentrations of TNF-alpha were higher in ADHF patients than in non-ADHF individuals. Yet, we found a moderate negative correlation between irisin and TNF-alpha, whereas an association between irisin and hs-CRP was mild. In addition to that, fasting glucose and HbA1c did not correlate with irisin at the baseline of the study. To note, hs-CRP was previously found to be an independent predictor of cardiovascular death in T2DM patients regardless of HF presentation [60]. However, derangements in adrenergic–adipokine signaling in a case of a deficiency of irisin production may be more valuable for adverse cardiac remodeling and cardiovascular outcomes among T2DM patients [64,65,66].

We suggested that differences in the levels of irisin and TNF-alpha at baseline in ADHF and non-ADHF was a result of particularities in the signature of cardiac and non-cardiac comorbidities along with therapy of HF. However, higher levels of NT-proBNP in ADHF patients when compared with non-ADHF individuals clearly indicated that there was a pre-existing risk of congestion due to certain conditions, which were able to increase the risks of hospitalization (arrhythmia, chronic kidney disease, and coronary artery disease). Indeed, ADHF patients presented higher AF, coronary artery disease, and dilated cardiomyopathy than non-ADHF. Moreover, this signature of comorbidities may explain the majority of cases of ADHF. In addition, clinical status in ADHF patients was respectively worse than those with non-ADHF, because IV NYHA class occurred frequently, but I NYHA class was detected rarely in ADHF compared to non-ADHF. Therefore, ADHF patients sufficiently differed from non-ADHF in LVEDS, LVMMI, and LAVI. Perhaps all these may explain why the baseline levels of NT-proBNP were higher in ADHF than in non-ADHF. Altogether, the results of our study confirmed that irisin has a unique capability to improve the prognostic information of NT-proBNP for ADHF and that it is a promising marker for serial monitoring.

## 5. Study Limitations

This study has several limitations. First, we included the patients with good control for T2DM who did not receive insulin. Consequently, we did not compare irisin levels and their predictive values for depending variables between T2DM and non-T2DM individuals. Second, the patients from both cohorts have been treated with guideline-recommended therapy and the majority of them received antagonists of the renin–angiotensin–aldosterone system in combination with beta-blockers and SGLT2 inhibitors. Third, we did not provide continuous monitoring of the biomarkers with the aim of clearly elucidating the dynamics of them. Perhaps this is an aim for investigations in the future. Yet, we had no possibilities to extend the discovery over other potential conditions affecting the risks of hospitalizations including iron deficiency, depression, missed drug intake, and the use of prohibited drugs, such as non-steroidal anti-inflammatory agents and appetite suppressants. We believe that these limitations would not be serious arguments against the interpretation of the study results.

## 6. Conclusions

We established that in T2DM patients with concomitant chronic HF, serum irisin levels were associated with the risk of ADHF. Yet, irisin added a discriminatory value to NT-proBNP for ADHF in this population. Additionally, T2DM patients with levels of irisin ≥ 7.85 ng/mL demonstrated benefits in clinical outcomes related to ADHF than those with <7.85 ng/mL. This finding may open a new prospective for the prediction of ADHF in T2DM patients with chronic HF regardless of the levels of NT-proBNP.

## Figures and Tables

**Table 1:** Baseline general characteristics of eligible HF patients.

Variables	Entire Chronic HF Patient Cohort (*n* = 480)	Patients with ADHF (*n* = 106)	Patients without ADHF (*n* = 374)	*p* Value
Demographics and anthropomorphic parameters				
Age, year	53 (40–67)	55 (46–67)	51 (41–63)	0.062
Male/female *n* (%)	272 (56.7)/208 (43.3)	62 (58.4)/44 (41.6)	210 (56.2)/164 (43.8)	0.124
BMI, kg/m^2^	25.6 ± 2.78	25.8 ± 2.60	25.3 ± 2.52	0.782
Waist circumference, cm	96.7 ± 3.90	97.7 ± 3.70	95.3 ± 3.36	0.420
WHR, units	0.88 ± 0.07	0.89 ± 0.08	0.85 ± 0.04	0.823
Comorbidities and CV risk factors				
Dyslipidemia, *n* (%)	385 (80.2)	84 (79.2)	301 (80.5)	0.781
Hypertension, *n* (%)	307(64.0)	71 (66.9)	236 (63.1)	0.643
Stable CAD, *n* (%)	166 (34.6)	42 (42.5)	124 (33.2)	0.024
DCM, *n* (%)	19 (4.0)	6 (5.7)	13 (3.5)	0.042
AF, *n* (%)	105 (21.9)	47 (44.3)	58 (15.5)	0.001
Paroxysmal/persistent AF, *n* (%)	56 (11.7)	29 (27.3)	27 (7.2)	0.001
Permanent AF, *n* (%)	49 (10.2)	18 (17.0)	31 (8.3)	0.012
Smoking, *n* (%)	196 (40.8)	42 (39.6)	154 (41.2)	0.860
Abdominal obesity, *n* (%)	215 (44.8)	48 (45.3)	167 (44.7)	0.823
LV hypertrophy, *n* (%)	382 (79.5)	85 (80.2)	297 (79.4)	0.837
CKD 1–3 grades, *n* (%)	118 (24.6)	39 (36.8)	79 (21.1)	0.012
HF phenotypes				
HFpEF, *n* (%)	212 (44.2)	40 (37.7)	172 (46.0)	0.056
HFmrEF, *n* (%)	156 (32.5)	35 (33.0)	121 (32.4)	0.523
HFrEF, *n* (%)	112 (23.3)	31 (29.2)	81 (21.7)	0.073
I/II HF NYHA class, *n* (%)	281 (58.6)	54 (50.9)	227 (60.7)	0.024
III HF NYHA class, *n* (%)	144 (30.0)	35 (33.1)	109 (29.1)	0.468
IV HF NYHA class, *n* (%)	55 (11.4)	17 (16.0)	38 (10.2)	0.001
Hemodynamics performances				
SBP, mm Hg	133 ± 8	132 ± 6	136 ± 7	0.621
DBP, mm Hg	77 ± 7	74 ± 5	79 ± 6	0.634
LVEDV, mL	162 (139–178)	168 (158–179)	160 (136–177)	0.442
LVESV, mL	88 (59–97)	93 (82–103)	84 (57–94)	0.012
LVEF, %	45 (34–57)	44 (32–55)	47 (36–67)	0.050
LVMMI, g/m^2^	138 ± 11	158 ± 13	129 ± 15	0.014
LAVI, mL/m^2^	41 (33–52)	45 (38–53)	39 (32–47)	0.010
E/e`, unit	11 ± 2	14 ± 3	13 ± 2	0.764
Biochemistry parameters				
eGFR, mL/min/1.73 m^2^	74 ± 9	63 ± 8	81 ± 9	0.026
HOMA-IR	6.95 ± 1.9	7.76 ± 2.9	6.12± 1.9	0.524
Fasting glucose, mmol/L	6.20 ± 1.2	6.81 ± 1.5	6.03 ± 1.3	0.642
HbA1c, %	6.40 ± 0.14	6.52 ± 0.12	6.25 ± 0.15	0.758
Creatinine, µmol/L	98.7 ± 9.8	108.6 ± 11.5	77.4 ± 8.9	0.042
TC, mmol/L	5.90 ± 0.91	6.22 ± 0.80	5.74 ± 0.70	0.186
HDL-C, mmol/L	0.96 ± 0.15	0.97 ± 0.17	1.01 ± 0.15	0.48
LDL-C, mmol/L	3.10± 0.20	3.38 ± 0.10	2.80 ± 0.14	0.016
TG, mmol/L	1.52 ± 0.19	1.61 ± 0.12	1.39 ± 0.15	0.044
Biomarkers				
hs-CRP, mg/L	4.20 (2.51–7.10)	4.35 (2.92–7.17)	4.06 (2.41–6.37)	0.641
TNF-alpha, pg/mL	2.95 (1.66–3.82)	3.41 (2.79–4.02)	2.39 (1.51–3.03)	0.042
NT-proBNP, pmol/mL	1215 (562–2155)	1719 (980–2457)	1057 (570–2607)	0.044
Irisin, ng/mL	5.64 (3.80–7.53)	4.96 (3.14–6.85)	7.95 (5.73–9.16)	0.001
Concomitant medications				
ACEIs, *n* (%)	296 (61.7)	61 (57.5)	208 (55.6)	0.541
ARBs, *n* (%)	72 (15.0)	19 (18.0)	53 (14.2)	0.226
ARNI, *n* (%)	112 (23.3)	26 (27.4)	86 (23.0)	0.211
Beta-blockers, *n* (%)	427 (89.0)	89 (83.9)	338 (90.4)	0.052
Ivabradine, *n* (%)	93 (19.4)	13 (12.3)	80 (21.4)	0.001
Calcium channel blockers, *n* (%)	131 (27.3)	24 (22.6)	107 (28.6)	0.064
MRA, *n* (%)	147 (30.6)	35 (33.0)	112 (29.9)	0.052
Loop diuretics, *n* (%)	383 (79.8)	76 (71.7)	307 (82.1)	0.014
Antiplatelet, *n* (%)	166 (34.6)	39 (36.8)	127 (34.0)	0.348
Anticoagulants, *n* (%)	105 (21.9)	47 (44.3)	58 (15.5)	0.001
Metformin, *n* (%)	480 (100.0)	106 (100.0)	374 (100.0)	1.000
SGLT2 inhibitors, *n* (%)	429 (89.4)	92 (86.8)	337 (90.1)	0.218
Statins, *n* (%)	453 (94.4)	93 (87.7)	360 (96.3)	0.048

Notes: Data of variables are given as mean (M) ± SD and median (25–75% interquartile range). Chi-square test was used to compare categorical variables. Student’s *t*-tests or ANOVA with Mann–Whitney U test were used to compare continuous variables between groups depending on variable distribution. *p* value, a difference between patient cohorts. Abbreviations: ACEIs, angiotensin-converting enzyme inhibitors; ARBs, angiotensin-II receptor blockers; ARNI, angiotensin receptor neprilysin inhibitors; BMI, body mass index; CAD, coronary artery disease; CKD, chronic kidney disease; DCM, dilated cardiomyopathy; DBP, diastolic blood pressure; E/e`, early diastolic blood filling to longitudinal strain ratio; GFR, glomerular filtration rate; HDL-C, high-density lipoprotein cholesterol; hs-CRP, high-sensitivity C-reactive protein; HFpEF, heart failure with preserved ejection fraction; HFmrEF, heart failure with mildly reduced ejection fraction; HFrEF, heart failure with reduced ejection fraction; LVEDV, left ventricular end-diastolic volume; LVESV, left ventricular end-systolic volume; LVEF, left ventricular ejection fraction; LVMMI, left ventricle myocardial mass index, left atrial volume index, LAVI; left atrial volume index; LDL-C, low-density lipoprotein cholesterol; MRA, mineralocorticoid receptor antagonist; NT-proBNP, N-terminal brain natriuretic pro-peptide; SBP, systolic blood pressure; SGLT2, sodium glucose linked transporter 2; TG, triglycerides; TC, total cholesterol; TNF-alpha, tumor necrosis factor-alpha; WHR, waist-to-hip ratio.

**Table 2:** Comparison of variables between baseline and 52 weeks after the administration of dapagliflozin.

Variables	Patient Groups	Baseline	52 weeks	Δ%	*p* Value
BMI, kg/m^2^	Entire group	25.6 ± 2.78	24.3 ± 1.92	−5.10	0.52
	ADHF	25.8 ± 2.60	25.6 ± 2.90	−0.80	0.76
	Non-ADHF	25.3 ± 2.52	23.8 ± 2.47	−5.90	0.13
SBP, mm Hg	Entire group	133 ± 8	129 ± 6	−3.00	0.46
	ADHF	132 ± 6	130 ± 5	−1.52	0.52
	Non-ADHF	136 ± 7	131 ± 6	−3.68	0.42
DBP, mm Hg	Entire group	77 ± 7	75 ± 5	−2.60	0.42
	ADHF	74 ± 5	73 ± 6	−1.40	0.48
	Non-ADHF	79 ± 6	76 ± 5	−3.80	0.44
LVEDV, mL	Entire group	162 (139–178)	160 (150–167)	−1.20	0.54
	ADHF	168 (158–179)	170 (156–182)	+1.20	0.18
	Non-ADHF	160 (136–177)	156 (135–171)	−2.50	0.14
LVESV, mL	Entire group	88 (59–97)	82 (78–86)	−6.90	0.04
	ADHF	93 (82–103)	92 (80–101)	−1.12	0.56
	Non-ADHF	84 (57–94)	78 (55–92)	−7.10	0.04
LVEF, %	Entire group	45 (34–57)	49 (44–55)	+8.80	0.05
	ADHF	44 (32–55)	45 (31–57)	+2.27	0.38
	Non-ADHF	47 (36–67)	52 (38–69)	10.60	0.05
LVMMI, g/m^2^	Entire group	138 ± 11	141 ± 5	+2.20	0.64
	ADHF	158 ± 13	162 ± 11	+2.50	0.36
	Non-ADHF	129 ± 15	130 ± 13	+0.80	0.82
LAVI, mL/m^2^	Entire group	41 (33–52)	40 (34–47)	−2.40	0.61
	ADHF	45 (38–53)	46 (40–52)	+2.20	0.43
	Non-ADHF	39 (32–47)	37 (33–42)	−5.12	0.14
E/e’, unit	Entire group	11 ± 2.0	10 ± 1.5	−9.00	0.52
	ADHF	14 ± 3	15 ± 4	+7.10	0.62
	Non-ADHF	13 ± 2	11 ± 3	−15.40	0.16
eGFR, mL/min/1.73 m^2^	Entire group	74 ± 9.0	76 ± 3.0	+2.70	0.46
	ADHF	63 ± 8	59 ± 5	−6.30	0.05
	Non-ADHF	81 ± 9	89 ± 6	+8.60	0.24
Fasting glucose, mmol/L	Entire group	6.20 ± 1.2	5.72 ± 1.1	−7.74	0.28
	ADHF	6.81 ± 1.5	6.89 ± 1.4	+1.00	0.64
	Non-ADHF	6.03 ± 1.3	5.43 ± 1.5	−10.0	0.24
HbA1c, %	Entire group	6.40 ± 0.14	6.47 ± 0.03	−1.74	0.31
	ADHF	6.52 ± 0.12	6.64 ± 0.15	+1.90	0.12
	Non-ADHF	6.25 ± 0.15	6.09 ± 0.13	−2.24	0.76
Creatinine, µmol/L	Entire group	98.7 ± 9.8	114.7 ± 7.5	+13.90	0.20
	ADHF	108.6 ± 11.5	138.2 ± 14.1	+21.70	0.04
	Non-ADHF	77.4 ± 8.9	80.5 ± 7.5	+3.90	0.72
hs-CRP, mg/L	Entire group	4.20 (2.51–7.10)	4.82 (2.39–7.31)	+13.1	0.026
	ADHF	4.35 (2.92–7.17)	5.70 (3.44–8.20)	+23.60	0.042
	Non-ADHF	4.06 (2.41–6.37)	3.85 (2.27–5.17)	−5.17	0.052
TNF-alpha, pg/mL	Entire group	2.95 (1.66–3.82)	2.97 (1.70–3.90)	+0.70	0.72
	ADHF	3.41 (2.79–4.02)	3.67 (2.90–4.22)	+7.60	0.18
	Non-ADHF	2.39 (1.51–3.03)	2.21 (1.38–3.01)	−7.50	0.042
NT-proBNP, pmol/mL	Entire group	1215 (562–2155)	1296 (672–1935)	+6.60	0.10
	ADHF	1719 (980–2457)	2142 (1170–3275)	+24.60	0.02
	Non-ADHF	1057 (570–2607)	887 (460–1215)	−16.1	0.04
Irisin, ng/mL	Entire group	5.64 (3.80–7.53)	5.81 (4.20–7.22)	+3.00	0.05
	ADHF	4.96 (3.14–6.85)	4.15 (2.83–5.58)	−0.81	0.26
	Non-ADHF	7.95 (5.73–9.16)	8.22 (6.40–10.12)	+3.30	0.04

Notes: Data of variables are given as mean ± SD and median (25–75% interquartile range). Abbreviations: DBP, diastolic blood pressure; E/e’, early diastolic blood filling to longitudinal strain ratio; GFR, glomerular filtration rate; hs-CRP, high-sensitivity C-reactive protein; HFpEF, heart failure with preserved ejection fraction; HFmrEF, heart failure with mildly reduced ejection fraction; HFrEF, heart failure with reduced ejection fraction; LVESV, left ventricular end-systolic volume; LVEF, left ventricular ejection fraction; LVMMI, left ventricle myocardial mass index, left atrial volume index, LAVI; left atrial volume index; NT-proBNP, N-terminal brain natriuretic pro-peptide; TNF-alpha, tumor necrosis factor-alpha.

**Table 3:** Predictors for ADHF in T2DM patients with chronic HF. The results of the univariate and multivariate log regression analysis.

Variables	Dependent Variable: ADHF					
	Univariate Log Regression			Multivariate Log Regression		
	OR	95% CI	*p*-Value	OR	95% CI	*p*-Value
Irisin (<7.85 ng/mL vs. ≥7.85 ng/mL)	1.24	1.08–1.46	0.001	1.20	1.08–1.45	0.001
NT-proBNP (>1215 pmol/mL vs. ≤1215 pmol/mL)	1.16	1.03–1.37	0.001	1.18	1.02–1.35	0.001
TNF-alpha (>2.95 pg/mL vs. ≤2.95 pg/mL)	1.06	1.02–1.11	0.012	1.05	1.00–1.08	0.112
hs-CRP (> 4.2 mg/L vs. ≤4.2 mg/L)	1.04	1.00–1.10	0.064	-		
eGFR (<74 mL/min/1.73 m^2^ vs. ≥74 mL/min/1.73 m^2^).	0.94	0.86–1.10	0.924	-		
LV hypertrophy (presence vs. absent)	1.10	1.01–1.18	0.044	1.05	1.00–1.12	0.124
AF (presence vs. absent)	1.12	1.00–1.25	0.066	-		
E/e’ (>11 units vs. ≤11 units)	1.03	0.99–1.06	0.682	-		
LVEF (<45% vs. ≥45%)	1.09	1.02–1.17	0.042	1.09	1.00–1.20	0.144
LAVI (>41 mL/m^2^ vs. ≤41 mL/m^2^)	1.12	1.03–1.21	0.026	1.07	1.00–1.13	0.148
ARBs (presence vs. absent)	0.97	0.93–1.05	0.645	-		
ARNI (presence vs. absent)	0.95	0.91–1.01	0.144	-		
SGLT2i (presence vs. absent)	0.94	0.90–1.02	0.268	-		

Abbreviations: AF, atrial fibrillation; ARBs, angiotensin-II receptor blockers; ARNI, angiotensin receptor neprilysin inhibitor; ACEIs, angiotensin-converting enzyme inhibitors; CI, confidence interval; eGFR, estimated glomerular filtration rate; E/e’, early diastolic blood filling to longitudinal strain ratio; NT-proBNP, N-terminal brain natriuretic pro-peptide; LVEF, left ventricular ejection fraction; LAVI; left atrial volume index; SGLT2i, sodium-glucose co-transporter 2-inhibitors; OR, odds ratio; TNF-alpha, tumor necrosis factor alpha.

**Table 4:** The comparisons of predictive models for ADHF.

Predictive Models	Dependent Variable: ADHF					
	AUC		NRI		IDI	
	M (95% CI)	*p* Value	M (95% CI)	*p* Value	M (95% CI)	*p* Value
Model 1 (NT-proBNP > 1215 pg/mL)	0.840 (0.791–0.865)	-	Reference	-	Reference	-
Model 2 (irisin < 7.85 ng/mL)	0.869 (0.800–0.937)	0.001	0.68 (0.63–0.72)	0.012	0.57 (0.53–0.62)	0.014
Model 3 (NT-proBNP > 1215 pg/mL + irisin < 7.85 ng/mL)	0.872 (0.808–0.942)	0.001	0.69 (0.65–0.74)	0.001	0.63 (0.59–0.68)	0.001

Abbreviations: AUC, area under curve; NT-proBNP, N-terminal brain natriuretic pro-peptide; HF, heart failure; IDI, integrated discrimination indices; NRI, net reclassification improvement. Note: *p* value indicates a significant difference in terms of Model 1.

## Data Availability

Not applicable.
